# Genetic and phenotypic correlations among feed efficiency, immune and production traits in indigenous chicken of Kenya

**DOI:** 10.3389/fgene.2022.1070304

**Published:** 2023-01-05

**Authors:** Sophie A. Miyumo, Chrilukovian B. Wasike, Evans D. Ilatsia, Jorn Bennewitz, Mizeck G. G. Chagunda

**Affiliations:** ^1^ Department of Animal Breeding and Husbandry in the Tropics and Sub-Tropics, University of Hohenheim, Stuttgart, Germany; ^2^ Livestock Efficiency Enhancement Group (LEEG), Department of Animal and Fisheries Sciences, Maseno University, Maseno, Kenya; ^3^ Kenya Agricultural and Livestock Research Organization, Naivasha, Kenya; ^4^ Department of Animal Breeding and Genetics, University of Hohenheim, Stuttgart, Germany

**Keywords:** indigenous chicken, feed efficiency, immunity, production, genetic parameters

## Abstract

This study aimed at estimating genetic and phenotypic relationships among feed efficiency, immune and production traits measured pre- (9–20 weeks of age) and post- (12 weeks from on-set of lay) maturity. Production traits were average daily gain (ADG) and average daily feed-intake (ADFI_1_) in the pre-maturity period and age at first egg (AFE), average daily feed-intake (ADFI_2_) and average daily egg mass (EM) in the post-maturity period. Feed efficiency comprised of residual feed intake (RFI) estimated in both periods. Natural antibodies binding to keyhole limpet hemocyanin (KLH-IgM) and specific antibodies binding to Newcastle disease virus (NDV-IgG) measured at 16 and 28 weeks of age represented immune traits pre- and post-maturity, respectively. In the growing period, 1,820 records on ADG, KLH-IgM and NDV-IgG, and 1,559 records on ADFI_1_ and RFI were available for analyses. In the laying period, 1,340 records on AFE, EM, KLH-IgM and NDV-IgG, and 1,288 records on ADFI_2_ and RFI were used in the analyses. Bi-variate animal mixed model was fitted to estimate (co)variance components, heritability and correlations among the traits. The model constituted sex, population, generation, line and genotype as fixed effects, and animal and residual effects as random variables. During the growing period, moderate to high heritability (0.36–0.68) was estimated for the production traits and RFI while the antibody traits had low (0.10–0.22) heritability estimates. Post-maturity, the production traits and RFI were moderately (0.30–0.37) heritable while moderate to high (0.25–0.41) heritability was estimated for the antibody traits. Genetic correlations between feed efficiency and production traits in both periods showed that RFI had negative genetic correlations with ADG (−0.47) and EM (−0.56) but was positively correlated with ADFI_1_ (0.60), ADFI_2_ (0.74) and AFE (0.35). Among immune and production traits, KLH-IgM and NDV-IgG had negative genetic correlations with ADG (−0.22; −0.56), AFE (−0.39; −0.42) and EM (−0.35; −0.16) but were positively correlated with ADFI_1_ (0.41; 0.34) and ADFI_2_ (0.47; 0.52). Genetic correlations between RFI with KLH-IgM (0.62; 0.33) and NDV-IgG (0.58; 0.50) were positive in both production periods. Feed intake, RFI and antibody traits measured in both production periods were positively correlated with estimates ranging from 0.48 to 0.82. Results from this study indicate selection possibilities to improve production, feed efficiency and immune-competence in indigenous chicken. The genetic correlations suggest that improved feed efficiency would be associated with high growth rates, early maturing chicken, high egg mass and reduced feed intake. In contrast, improved general (KLH-IgM) and specific (NDV-IgG) immunity would result in lower growth rates and egg mass but associated with early sexual maturation and high feed intake. Unfavorable genetic correlations between feed efficiency and immune traits imply that chicken of higher productivity and antibody levels will consume more feed to support both functions. These associations indicate that selective breeding for feed efficiency and immune-competence may have genetic consequences on production traits and should therefore be accounted for in indigenous chicken improvement programs

## Introduction

Indigenous chicken (*Gallus gallus domesticus*) play significant roles in nutrition, food and income security in many rural households in most countries in the tropical regions (Alders and Pym, 2009). In Kenya, indigenous chicken (IC) account for about 80% of the total chicken population and are kept by over 75% of the rural households (KNBS, 2019). Their popularity, particularly among rural households, is attributed to their ability to produce under low input systems and adapt to local environmental conditions (Olwande et al., 2010). Despite their adaptive ability, IC are predominantly raised under challenging environments which limit their optimal utilization. Seasonal variation in availability and quality of feed resources is a major challenge that limit productivity (Miyumo et al., 2018). Given that feed represents about 60%–70% of the total production costs, increased feed costs directly affect profitability of the enterprise (Besbes, 2009). Under low input systems, IC are exposed to a myriad of pathogens that cause various diseases which result in massive production and economic losses (Okeno et al., 2011). Of significance is the Newcastle disease (NCD) which is endemic among chicken in the tropics and is reported to be a major factor limiting productivity due to high prevalence and mortality rates (Lwelamira, 2012). These environmental conditions are expected to negatively influence the availability and quality of feed resources and pathogen epidemiology due to climate change impacts.

Genetic improvement of feed efficiency provides an avenue to select individuals that are able to efficiently convert available feed resources into products and support their maintenance requirements. Besides, selection for feed efficient animals contributes to reduced feed costs and nitrogenous wastes, and minimizes the environmental foot-print (Moore et al., 2009; Zhang and Aggrey, 2003). Measures such as residual feed intake (RFI) and feed conversion ratio (FCR) have extensively been used to improve feed efficiency in chicken (Prakash et al., 2020), swine (Patience et al., 2015) and cattle (Berry and Crowley, 2012) due to their moderate to high heritability. Selective breeding for antibody traits related to general and specific immune-competence provide an opportunity to enhance disease resistance in animals (Cheng et al., 2013). Natural antibodies binding to keyhole limpet hemocyanin (KLH-IgM) antigen in chicken have previously been used to improve general disease resistance (Berghof et al., 2019) and are also associated with higher survival rates (Sun et al., 2013). On the other hand, specific antibodies binding to the NCD virus (NDV-IgG) have been found heritable and able to confer specific disease resistance against NCD (Lwelamira, 2012).

The Kenyan IC is a highly diverse population with a large plasticity in genomic regions that confer adaptability to challenging production conditions, poor nutrition and tolerance to pathogens (Ngeno et al., 2015). Such genetic advantage could be exploited to improve feed efficiency and immune-competence in this population. However, in cases of resource scarcity, trade-offs between different functions in the body may be expected when a negative dependency between resource acquisition and resource allocation exists (Zerjal et al., 2021). Even in environments where *ad libitum* feed resources are available, Rauw (2012) indicates that there is a limited amount of feed an individual can ingest, assimilate, use, and share among various functions in the body with preferential partitioning of resources to functions of interest. For instance, divergent selection for chicken lines of high growth rates (van der Most et al., 2011) or early maturing chicken lines with high egg numbers (Pinard-van der Laan et al., 1998) resulted in lower antibody responses upon disease challenge. Conversely, in a high egg laying line, van der Klein et al. (2015) estimated a positive genetic correlation between feed efficiency and natural antibodies indicating that immune-competent chicken required more feed to support both high maintenance and production requirements. These studies demonstrate competitive nutrient and energy partitioning between production and immune functions, and also suggest involvement of pleiotropic gene action among feed use efficiency, production and immune functions.

Understanding the nature of trade-offs between production and immune traits based on available feed resources is crucial when breeding for a robust chicken that is capable of high productivity while maintaining its adaptive potential to stressors in its production environment. Considering the ability of IC to produce and survive in poor conditions, studies on the impact of improving their resource use efficiency and immune-competence on growth and egg production are sparse. The current study aimed at estimating genetic and phenotypic correlations among feed efficiency, antibody and production traits measured pre- and post-maturity in indigenous chicken. Findings from this study would provide information on the genetic background of feed efficiency, antibody and production traits and the pleiotropic nature among the traits that could be applied in chicken breeding, especially when utilizing highly diverse populations as genetic resources.

## Materials and methods

### Experimental population

The study was conducted at the Non-Ruminant Research Institute of the Kenya Agriculture and Livestock Research Organization (NRI-KALRO) in Naivasha-Kenya. Two populations of chicken exist at the research station; an indigenous chicken (IC) population and a synthetic breed population known as KALRO chicken (KC). The IC population comprises of ecotypes from various agro-ecological zones grouped into three phylogenetic clusters based on major histocompatibility complex (MHC) linked microsatellite markers (Ngeno et al., 2015). Cluster one constitutes ecotypes from the Western and South-Rift regions that exhibit warm and humid weather; cluster two constitutes ecotypes from the North-Rift and North-Eastern regions that are considered arid and semi-arid; and cluster three constitute ecotypes from the Coastal region that is hot and humid. Within the clusters seven genetic groups exist namely normal feathered, naked neck, frizzled feathered, crested head, feathered shanks, dwarf and game-gaited (*Kuchi*) structure (Magothe et al., 2012). The synthetic (KC) population, on the other hand, originated from a dual-purpose hybrid that was subjected to a systematic and continuous *inter-se* mating resulting to highly segregated individuals in subsequent generations (Ilatsia et al., 2017). Based on plumage dominance, two distinct groups were isolated; black and white barred plumage (KC1) and black plumage (KC2). The groups were subsequently subjected to within line mating to stabilize the respective plumage colour.

The two populations are under continuous selection to develop meat (ML) and egg (EL) lines (Ilatsia et al., 2017). The ML birds are selected for body weight at 12 weeks of age (BW_12_) (Ngeno et al., 2013). The EL are selected based on age at first egg (AFE) (Dana et al., 2011). The chicken breeding program at NRI-KALRO is in its initial stages with a small population size and limited number of pedigree and performance records, therefore, selection for ML and EL is based on phenotypic information (Ilatsia et al., 2017). The selection criterion involves retaining males and females whose phenotypic BW_12_ is at least one standard deviation above average as meat lines. Chicken whose phenotypic BW_12_ is below average are considered for AFE evaluation. This involves assessing females using own phenotypic AFE records while males are assessed based on average phenotypic information on AFE from their respective daughters and dams. Individuals with below average AFE are retained. In this study, three generations (generations four to six) of these chicken comprising of both ML and EL were considered. A total of 1820 chicken were included in the study.

Management of the experimental population followed standard operating procedures of the breeding program at NRI-KALRO (Ilatsia et al., 2017). Birds were fed a starter ration (20% CP and 2800 Kcal ME/Kg) from day one to week 8 of age, growers ration (18% CP and 2750 Kcal ME/Kg) from week 8 to week 19 of age and layers ration (16% CP and 2600 Kcal ME/Kg) from week 20 of age to the rest of the test period. Routine health management involved vaccination against endemic diseases namely Marek’s disease (MD), Newcastle disease (NCD), and infectious bursal disease (IBD). In addition, experimental chickens were vaccinated against fowl pox (week 6) and fowl typhoid (week 18), and dewormed and disinfected routinely.

### Data collection

Ethical approval to conduct the study was provided by the Institutional Animal Care and Use Committee (IACUC) of KALRO - Veterinary Science Research Institute (VSRI) (KALROVSRI/IACUC019/30082019). Measurement of antibody traits during the growing period and laying period involved blood sampling of the experimental chicken *via* the wing vein at the ages of 16 and 28 weeks, respectively. These age points were considered based on the average age at point of maximum growth rate (16 weeks) and sexual maturity (28 weeks; age at first egg for the females and extended to their respective male relatives) of the third generation. Blood sampling procedure was carried out without anesthesia and no chicken was killed for sample collection. Post sampling, birds were given multi-vitamins and observed for any post-trauma effects. Plasma was extracted from the blood samples for further use in antibody measurement.

Natural antibodies binding to keyhole limpet hemocyanin (KLH-IgM) and specific antibodies binding to Newcastle disease (NCD) virus (NDV-IgG) were used to measure general disease resistance and specific resistance against NCD, respectively. The KLH antigen was considered due to its suitability to measure natural antibodies while NDV antigen was considered due to the negative impact of NCD on IC population (Lwelamira, 2012; Sun et al., 2013). Titers of KLH-IgM isotype was determined using an indirect two-step ELISA (Enzyme-linked Immunosorbent assay) as described by Berghof et al. (2015). Similarly, titers of NDV-IgG isotype were measured using indirect ELISA as described by Bell and Lelenta (2002). Absorbance levels were measured at 450 nm (reference wavelength at 620 nm) using a spectrophotometer ELISA reader (mrc Scientific Instrument-UT-6100, Israel). Pre-defined serial standard dilutions of the antibody traits (Bell and Lelenta, 2002; Berghof et al., 2015) and their respective absorbance reads were used to obtain standard curves by fitting a four-parameter logistic model (Herman et al., 2008) using GraphPad Prism 9.1 (GraphPad Software). Subsequently, concentration levels of the antibody traits in plasma samples were calculated from the standard curves using their respective absorbance reads. The concentration levels of the antibody traits were thereafter adjusted to their respective sample dilution factors (1:10 for KLH-IgM and 1:40 for NDV-IgG) and expressed as log_2_ values. This was done separately for each plate to partly correct for plate differences and allow values to be comparable across plates.

Residual feed intake (RFI) and feed conversion ratio (FCR) were used to assess feed efficiency during the growing and early laying phases. To estimate the efficiency measures, average daily feed intake (ADFI), average daily gain (ADG) and metabolic body weight (MBW) were considered during the growers’ phase (from 8–20 weeks of age). At week eight of age, birds were weighed (initial weight), transferred to individual feeding pens and allowed a 1-week acclimatization period. Thereafter, daily feed intake recording commenced from week 9 and ended at week 20, upon which birds were weighed (final weight) again. Average daily gain was obtained as the difference between final and initial body weight divided by the test period. Cumulative feed intake from week 9 to week 20 was divided by the duration of test to obtain ADFI. Metabolic body weight was calculated based on the average between initial and final body weights raised to the power of 0.75 (BW^0.75^). During the laying period, ADFI, average daily egg mass (EM), MBW and ADG were considered. At the onset of lay, initial body weight was measured, daily feed intake, egg number and weight recording commenced and continued until week 12, at which final body weight was measured. Estimation of ADG, ADFI and MBW were made as described in the growing period. Egg number was summed and egg weights averaged during the 12 weeks of test period. Thereafter, EM was calculated as the product of total egg number and average egg weight divided by the test period (12 weeks) (Yuan et al., 2015). Feed conversion ratio was estimated as a ratio of ADFI and ADG in the growing period and as a ratio of ADFI and EM in the laying period. Residual feed intake was computed as the difference between observed ADFI and expected ADFI. Observed ADFI is actual measurement taken on feed intake while expected ADFI is feed intake predicted on basis of MBW and production traits.

Random effect models were fitted to compute RFI using Eq. 1 (growing period) and Eq. 2 (laying period) using the *lme4* package of R Software (R Core Team, 2021). The model allows parameters to vary between- and within-individuals hence, improves the accuracy of predicting feed intake and also aids in selection when confronted with birds with similar RFI values (Aggrey and Rekaya, 2013).

$${RFI}_{i}=Y_{i}-\left(b_{0}+b_{1}{ADG}_{i}+b_{2}{MBW}_{i}+\alpha_{1i}{ADG}_{i}+\alpha_{2i}{MBW}_{i}\right)$$ (1)

$${RFI}_{i}=Y_{i}-\left(b_{0}+b_{1}{EM}_{i}+b_{2}{ADG}_{i}+b_{3}{MBW}_{i}+\alpha_{1i}{EM}_{i}+\alpha_{2i}{ADG}_{i}+\alpha_{3i}{MBW}_{i}\right)$$ (2)

Where: RFI_i_ is the estimated residual feed intake for ith bird; Y_i_ is the average daily feed intake record of ith bird; ADG_i_, MBW_i_ and EM_j_ are the observed average daily gain, metabolic body weight and average daily egg mass of ith bird, respectively; b_k_ are the fixed regression coefficients (k = 0, 1, 2, 3) related to the population; α_ki_ is the random regression coefficient (k = 1, 2, 3) specific to i^th^ bird for the observed traits and assumed to have ∼ N (0, σ^2^α) distribution.

Production traits included data on the traits under selection in the study population; BW_12_ and AFE. In addition, during the growing period, ADG, MBW, and ADFI were considered. In the laying period, body weight at first egg (BW_AFE_), egg weight of first egg (EW_AFE_), ADG, MBW, ADFI, EM, cumulative egg number (EN_12_) and average daily egg weight (EW_12_) 12 weeks post on-set of lay were considered.

### Statistical analysis

A series of bi-variate animal models (Eq. 3) were fitted to estimate genetic and phenotypic parameters for antibody, feed efficiency and production traits by restricted maximum likelihood through the average information (AI-REML) algorithm of the WOMBAT software (Meyer, 2007).

$$\left[\begin{array}{c} y_{i}\\ y_{j} \end{array}\right]=\left[\begin{array}{cc} X_{i} & 0\\ 0 & X_{j} \end{array}\right]\left[\begin{array}{c} b_{i}\\ b_{j} \end{array}\right]+\left[\begin{array}{cc} Z_{ii} & Z_{ij}\\ Z_{ji} & Z_{jj} \end{array}\right]\;\left[\begin{array}{c} a_{i}\\ a_{j} \end{array}\right]+\left[\begin{array}{c} e_{i}\\ e_{j} \end{array}\right]$$ (3)

where: **y**_i_ is the vector of observations for the antibody, feed efficiency and production traits (considering two at a time); **b**_**i**_ the vector of fixed effects; **a**_**i**_ the vector of random animal additive genetic effects assumed to be **a** ∼ N (0, **A**σ^2^a) in which **A** is the numerator relationship matrix and σ^2^a is the additive genetic variance; **e**_**i**_ the vector of random residual effect assumed to be **e** ∼ N (0, **I**σ^2^e) in which **I** is an identity matrix and σ^2^e is the residual variance; **X**_i_, and **Z**_i_ are incidence matrices relating records to fixed and random animal effects, respectively. The pedigree used to construct the numerator relationship matrix consisted of 2,013 individuals (inclusive of those with and without records) from three generations. Assumed covariance structures of the random model terms among the traits are presented below.

$$Var\;\left[\begin{array}{c} a_{i}\\ a_{j}\\ e_{i}\\ e_{j} \end{array}\right]=\left[\begin{array}{c} \begin{array}{cc} {\sigma_{\alpha }^{2}}_{ii}A & {\sigma_{\alpha }^{2}}_{ij}A\\ {\sigma_{\alpha }^{2}}_{ji}A & {\sigma_{\alpha }^{2}}_{jj}A \end{array}\;\begin{array}{cc} 0 & 0\\ 0 & 0 \end{array}\\ \begin{array}{cc} 0 & 0\\ 0 & 0 \end{array}\;\begin{array}{cc} {\sigma_{e}^{2}}_{ii}I & {\sigma_{e}^{2}}_{ij}I\\ {\sigma_{e}^{2}}_{ji}I & {\sigma_{e}^{2}}_{jj}I \end{array} \end{array}\right]$$ (4)

where **a**_**i**_, **e**_**i**_, **A** and **I** were described in Eq. 3; σ^2^a_ii_ is the additive genetic variance for trait i; σ^2^a_ij_ is the additive genetic covariance between trait i and trait j; σ^2^e_ii_ is the residual variance for trait i; σ^2^e_ij_ is the residual covariance between trait i and trait j; zero covariance between additive genetic effect and residual effect was assumed.

From preliminary tests of fixed factors significantly (*p* < 0.05) affecting the traits, sex, population, generation, line and genotype were included in the bivariate analyses as fixed effects. Likelihood ratio test was used to determine whether genetic correlations among the traits were significantly different from zero, by comparing Eq. 3 to a bivariate model with additive genetic covariance fixed at zero. On the other hand, Fisher’s *r* to *z*-transformation was used to test whether phenotypic correlations were significantly different from zero using the following test statistic:

$$z=0.5\;\ln\frac{\left(1+r\right)}{\left(1-r\right)}$$

where r is the estimated phenotypic correlation, and z follows a normal distribution with standard deviation 1/√(n-3), where n is the sample size. Various combinations of traits in different bi-variate models fitted resulted in several estimates of variance components and variance ratios for each of the traits. Therefore, to obtain a global estimate for the genetic parameters on each of the traits, pooling was carried out by weighting estimates by the inverse of their respective sampling variance.

## Results

Descriptive statistics on production, feed efficiency and immune traits measured during the growing and laying periods are presented in Table 1. Mean estimates of production traits under selection in the study population showed that BW_12_ was 1205.81 g and AFE was 23 weeks. During the growing period, chicken had a higher growth rate of 13.82 g/d compared to the laying period (3.21 g/d). In contrast, mean estimates of ADFI, MBW and ADFI were higher in the laying period than in the growing period. At the on-set of lay, mean estimates of BW_AFE_ and EW_AFE_ were 1616.81 g and 42.30 g, respectively. Twelve weeks post on-set of lay showed that the study population laid on average 54 eggs with an average daily egg weight of 47.86 g resulting to an average daily egg mass of 28.87 g/d. In feed efficiency measures (RFI and FCR), chicken had higher mean estimates during the laying period (RFI = 0.05 g/d; FCR = 7.47 g:g) compared to the growing period (RFI = 0.01 g/d; FCR = 4.69 g:g). In immune traits, mean estimates of KLH-IgM in both production periods were higher than the NDV-IgG estimates. Between the two production periods, the laying period had higher mean estimates for the antibody traits than the growing period. The coefficient of variation of traits measured in both production periods indicated wide dispersion of estimates from their respective means.

**TABLE 1:** Number of observations (N), mean, standard deviation (SD) and coefficient of variation (CV) of production, feed efficiency and immune traits measured during the growing and laying periods.

Period^a^	Trait^b^	N	Mean	SD	CV (%)
Growing	BW_12_ (g)	1,820	1205.81	84.55	7.01
	ADG (g/d)	1,820	13.82	3.80	27.50
	MBW (g)	1,820	188.43	18.42	9.78
	ADFI (g/d)	1,559	95.16	4.17	4.38
	RFI (g/d)	1,559	0.01	4.14	41400.00
	FCR (g:g)	1,559	4.69	2.27	48.40
	KLH-IgM (ng/mL)	1,820	7.08	1.24	17.51
	NDV-IgG (ng/mL)	1,820	5.48	0.94	17.15
Laying	AFE (weeks)	1,340	23.00	2.88	12.52
	BW_AFE_ (g)	1,340	1616.81	175.47	10.85
	EW_AFE_ (g)	1,340	42.30	9.11	21.53
	ADG (g/d)	1,340	3.21	1.44	44.90
	MBW (g)	1,340	271.67	20.98	7.72
	EN_12_ (number)	1,340	54.00	15.6	28.89
	EW_12_ (g)	1,340	47.86	6.97	14.56
	EM_12_ (g/d)	1,340	28.87	9.95	34.46
	ADFI (g/d)	1,288	115.21	16.65	14.45
	RFI (g/d)	1,288	0.05	13.79	27580.00
	FCR (g:g)	1,288	7.47	2.9	38.82
	KLH-IgM (ng/mL)	1,340	12.33	0.91	7.38
	NDV-IgG (ng/mL)	1,340	11.22	0.67	5.97

^a^ Growing period was from 8 to 20 weeks of age; Laying period was 12 weeks from onset of lay.

^b^ BW_12_ is body weight at week 12; ADG, is average daily gain; MBW, is metabolic weight; ADFI, is average daily feed intake; RFI, is residual feed intake; FCR, is feed conversion ratio; KLH-IgM, is natural antibodies of IgM isotype binding to KLH, antigen; NDV-IgG, is specific antibodies of IgG binding to NDV, antigen; AFE, is age at first egg; BW_AFE_, is body weight at age at first egg; EW_AFE_, is egg weight at age at first egg; EN_12_ is cumulative number of eggs 12 weeks from onset of lay; EW_12_ is average egg weight 12 weeks from onset of lay; EM_12_ is average daily egg mass 12 weeks from onset of lay.

Variation due to additive genetic effects and residual effects, and heritability estimates are presented in Table 2. During the growing period, additive genetic effect contributed more to phenotypic variation of BW_12_, ADG, RFI and FCR than residual effects. In contrast, residual effects were higher on MBW, ADFI, KLH-IgM and NDV-IgG than additive genetic effects. Across the traits measured during the laying period, residual effect was more eminent on AFE, ADG, MBW, EN_12_, EM_12_, KLH-IgM, and NDV-IgG than additive genetic effect. On the other hand, phenotypic variation due to additive genetic effect was highest in BW_AFE_, EW_AFE_ and EW_12_ than additive genetic effects. Moderate to high heritabilities (0.32–0.68) were estimated for production traits (BW_12_, ADG, MBW, and ADFI) measured during the growing period. Among the feed efficiency measures, heritability estimates for RFI and FCR were 0.43 and 0.45, respectively. Low to moderate heritability was estimated in KLH-IgM (h^2^ = 0.22) and NDV-IgG (h^2^ = 0.10). At the onset of lay, heritability estimates for AFE, BW_AFE_ and EW_AFE_ were 0.31, 0.37 and 0.59, respectively. For egg production traits measured 12 weeks post onset of lay, heritability estimates for EN_12_, EW_12_ and EM_12_ were 0.23, 0.64, and 0.33, respectively. Contrary to the growing period, ADG (h^2^ = 0.02), MBW (h^2^ = 0.26) and ADFI (h^2^ = 0.37) measured during the laying period had lower heritability estimates. Feed efficiency measures in the laying period had lower heritability (RFI = 0.30; FCR = 0.34) estimates than in the growing period. Heritability estimates for KLH-IgM and NDV-IgG in the laying period were 0.41 and 0.25, respectively. These estimates were higher compared to those obtained for traits in the growing period.

**TABLE 2:** Estimates of additive genetic variance (σ_a_^2^)^1^, residual variance (σ_e_^2^)^1^ and heritability (h^2^)^1^ of production, feed efficiency and immune traits measured in growing and laying periods.

Period^b^	Trait^c^	σ^2^ _a_	σ^2^_e_	h^2^
Growing	BW_12_ (g)	34.77 ± 0.03	10.98 ± 0.03	0.44 ± 0.07
	ADG (g/d)	5.15 ± 0.94	2.38 ± 0.68	0.68 ± 0.10
	MBW (g)	85.61 ± 9.27	184.56 ± 5.62	0.32 ± 0.10
	ADFI (g/d)	17.45 ± 1.40	21.03 ± 1.18	0.36 ± 0.05
	RFI (g/d)	23.15 ± 0.04	10.45 ± 0.26	0.43 ± 0.07
	FCR (g:g)	2.54 ± 0.25	1.86 ± 0.32	0.45 ± 0.04
	KLH-IgM (ng/mL)	0.20 ± 0.08	0.34 ± 0.16	0.22 ± 0.03
	NDV-IgG (ng/mL)	0.24 ± 0.03	0.41 ± 0.34	0.10 ± 0.02
Laying	AFE (weeks)	0.37 ± 0.14	3.35 ± 0.36	0.31 ± 0.09
	BW_AFE_ (g)	979.78 ± 0.04	957 ± 0.04	0.37 ± 0.12
	EW_AFE_ (g)	70.12 ± 13.79	7.72 ± 9.94	0.59 ± 0.13
	ADG (g/d)	0.04 ± 0.14	1.75 ± 0.17	0.02 ± 0.08
	MBW (g)	107.39 ± 47.90	300.66 ± 44.88	0.26 ± 0.11
	EN_12_ (number)	41.55 ± 19.91	137.28 ± 19.14	0.23 ± 0.11
	EW_12_ (g)	25.11 ± 5.70	8.64 ± 4.33	0.64 ± 0.14
	EM_12_ (g/d)	18.13 ± 6.75	36.85 ± 6.08	0.33 ± 0.12
	ADFI (g/d)	77.30 ± 28.31	133.66 ± 24.87	0.37 ± 0.13
	RFI (g/d)	61.73 ± 23.93	129.56 ± 21.86	0.30 ± 0.05
	FCR (g:g)	2.69 ± 0.82	5.51 ± 0.76	0.34 ± 0.04
	KLH-IgM (ng/mL)	0.62 ± 0.20	0.80 ± 0.04	0.41 ± 0.08
	NDV-IgG (ng/mL)	0.46 ± 0.02	0.50 ± 0.02	0.25 ± 0.06

^a^ ± SE, standard errors.

^b^ Growing period was from 8 to 20 weeks of age; Laying period was 12 weeks from onset of lay.

^c^ BW_12_ is body weight at week 12; ADG, is average daily gain; MBW, is metabolic weight; ADFI, is average daily feed intake; RFI, is residual feed intake; FCR, is feed conversion ratio; KLH-IgM, is natural antibodies of IgM isotype binding to KLH, antigen; NDV-IgG, is specific antibodies of IgG binding to NDV, antigen; AFE, is age at first egg; BW_AFE_, is body weight at age at first egg; EW_AFE_, is egg weight at age at first egg; EN_12_ is cumulative number of eggs 12 weeks from onset of lay; EW_12_ is average egg weight 12 weeks from onset of lay; EM_12_ is average daily egg mass 12 weeks from onset of lay.

Genetic (r_g_) and phenotypic (r_p_) correlations among production, feed efficiency and immune traits measured during the growing period are presented in Table 3. Genetic correlations among the production traits (BW_12_, ADG, MBW and ADFI) were highly positive (0.43–0.86; *p* < 0.01). Between feed efficiency and production traits, RFI was negatively correlated with BW_12_ (r_g_ = −0.66), ADG (r_g_ = −0.47) and MBW (r_g_ = −0.52) but positively correlated with ADFI (r_g_ = 0.60). On the other hand, FCR had positive (r_g_ = 0.44–0.49) correlations with BW_12_, MBW and ADFI but was highly negatively correlated with ADG (r_g_ = −0.79). Among antibody traits and production traits, negative correlations were estimated between KLH-IgM with BW_12_ (r_g_ = −0.47; *p* < 0.01) and between NDV-IgG with ADG (r_g_ = −0.56; *p* < 0.01). Between feed efficiency and immune measures, ADFI and RFI was positively correlated with KLH-IgM (r_g_ = 0.41–0.62) and NDV-IgG (r_g_ = 0.34–0.58). Positive genetic correlation was estimated between RFI and FCR (r_g_ = 0.51). Although non-significant, KLH-IgM and NDV-IgG had a negative genetic correlation. Phenotypic correlations among the traits followed a similar pattern as genetic correlations, apart from correlations between FCR with BW_12_, MBW, and RFI, and between RFI and BW_12_. Significant positive phenotypic correlations (0.44–0.82; *p* < 0.01) were estimated among the production traits. Residual feed intake had positive phenotypic correlation with BW_12_ (0.36; *p* < 0.001) while FCR was negatively (r_p_ = −0.42 to −0.94; *p* < 0.01) correlated to BW_12_, ADG and MBW but was positively correlated to ADFI (r_p_ = 0.88; *p* < 0.001). There was a positive (r_p_ = 0.22; *p* < 0.001) correlation between the two measures of feed efficiency. Positive phenotypic correlations were estimated between KLH-IgM with ADG, MBW, and ADFI.

**TABLE 3:** Estimates of genetic^a^ (lower diagonal) and phenotypic^a^ (upper diagonal) correlations among production, feed efficiency and immune traits measured during the growing period (9–20 weeks of age).

Traits^b^	BW_12_	ADG	MBW	ADFI	RFI	FCR	KLH-IgM	NDV-IgG
BW_12_		0.47 ± 0.04^***^	0.82 ± 0.01^***^	0.44 ± 0.04^***^	0.36 ± 0.09^***^	−0.42 ± 0.09^**^	0.04 ± 0.03	0.05 ± 0.03
ADG	0.43 ± 0.09^***c^		0.69 ± 0.02^***^	0.72 ± 0.04^***^	−0.03 ± 0.12	−0.94 ± 0.01^**^	0.18 ± 0.05^***^	−0.18 ± 0.05
MBW	0.75 ± 0.02^***^	0.86 ± 0.05^***^		0.61 ± 0.20^**^	−0.01 ± 0.14	−0.53 ± 0.03^***^	0.27 ± 0.06^***^	0.12 ± 0.06
ADFI	0.78 ± 0.05^***c^	0.58 ± 0.18^**^	0.44 ± 0.16^**^		0.05 ± 0.06	0.88 ± 0.06^***^	0.56 ± 0.22^**^	0.20 ± 0.18
RFI	−0.66 ± 0.18^*^	−0.47 ± 0.08^**^	−0.52 ± 0.12^**^	0.60 ± 0.15^**^		0.22 ± 0.04^***^	0.02 ± 0.06	0.04 ± 0.05
FCR	0.44 ± 0.18^*^	−0.79 ± 0.06	0.47 ± 0.14^***^	0.49 ± 0.16^***^	0.51 ± 0.11^**^		−0.18 ± 0.15	0.10 ± 0.08
KLH-IgM	−0.47 ± 0.15^**^	−0.22 ± 0.19	0.46 ± 0.17^**^	0.41 ± 0.13^**^	0.62 ± 0.17^***^	0.47 ± 0.34		0.01 ± 0.03
NDV-IgG	0.06 ± 0.66	−0.56 ± 0.11^**^	0.49 ± 0.15^***^	0.34 ± 0.09^**^	0.58 ± 0.13^***^	0.03 ± 0.57	−0.55 ± 0.38	

^a^ ± SE, standard errors.

^b^ BW_12_ is body weight at week 12 of age; ADG, is average daily gain; MBW, is metabolic weight; ADFI, is average daily feed intake; RFI, is residual feed intake; FCR, is feed conversion ratio; KLH-IgM, is natural antibody of IgM isotype binding to KLH, antigen measured at 16 weeks; NDV-IgG, is specific antibody IgG isotype binding to NDV, antigen measured at 16 weeks of age.

***p < 0.001, **p < 0.01, *p < 0.05.

Genetic (r_g_) and phenotypic (r_p_) correlations among production, feed efficiency and immune traits measured during the laying period are presented in Table 4; Table 5. Positive (0.44–0.67; *p* < 0.01) genetic correlations were estimated among egg-related traits (AFE, BW_AFE_ and EW_AFE_) measured at the onset of lay. Age at first egg had negative correlation EN_12_ (r_g_ = −0.54) but was positively correlated with EW_12_ (r_g_ = 0.74) and EM_12_ (r_g_ = 0.41). Body weight at sexual maturity was positively (r_g_ = 0.46–0.72) correlated with MBW, EW_12_, EM_12,_ and ADFI but was negatively correlated with ADG (r_g_ = −0.38) and EN_12_ (r_g_ = −0.59). Egg weight of first egg had positive correlation with EW_12_ (r_g_ = 0.76; *p* < 0.001). Positive (0.39–0.75) genetic correlations were estimated between MBW with ADG, EN_12_, EM_12_ and ADFI. Cumulative number of eggs 12 weeks’ post-onset of lay was negatively (r_g_ = −0.52) correlated with EW_12_ but was positively correlated with EM_12_ (r_g_ = 0.93) and ADFI (r_g_ = 0.40). Average daily egg weight had positive correlations with EM_12_ (r_g_ = 0.66) and ADFI (r_g_ = 0.61). Between feed efficiency and production traits, RFI was negatively (r_g_ = 0.44 to −0.61; *p* < 0.01) correlated with MBW, EN_12_ and EM_12_ but positively (r_g_ = 0.45 to 0.74; *p* < 0.001) correlated with EW_12_ and ADFI. Feed conversion ratio had positive (r_g_ = 0.51–0.82) correlations with MBW, EW_12_ and ADFI but had negative (r_g_ = −0.74 to −0.82) correlations with EN_12_ and EM_12_. Genetic correlations between immune and production traits showed that KLH-IgM was negatively (r_g_ = −0.35 to −0.43) correlated with AFE, BW_AFE_, EN_12_, EW_12_ and EM_12_ but was positively (r_g_ = 0.47–0.51) correlated with MBW and ADFI. Similarly, NDV-IgG was negatively (r_g_ = −0.35 to −0.49) correlated with AFE, BW_AFE_, EN_12_, EW_12_ and EM_12_ but was positively (r_g_ = 0.44–0.52) correlated to MBW and ADFI. Positive (0.33–0.50) genetic correlations were estimated between RFI and the antibody traits. Between RFI and FCR, positive (r_g_ = 0.41) genetic correlations were estimated. Similar to the growing period, although non-significant, KLH-IgM and NDV-IgG were negatively (r_g_ = −0.49) correlated. Although lower estimates, phenotypic correlations among production, feed efficiency and antibody traits followed a similar a pattern as the genetic correlations.

**TABLE 4:** Estimates of genetic^a^ (lower diagonal) and phenotypic^a^ (upper diagonal) correlations among production, feed efficiency and immune traits during the laying period (12 weeks from on-set of lay).

Traits^b^	AFE	BW_AFE_	EW_AFE_	ADG	MBW	EN_12_	EW_12_	EM_12_	ADFI
AFE		0.35 ± 0.04^***^	0.31 ± 0.24	−0.21 ± 0.19	0.28 ± 0.04^***^	−0.20 ± 0.05^*^	0.28 ± 0.04^***^	0.10 ± 0.05	0.04 ± 0.05
BW_AFE_	0.46 ± 0.12^*^		0.13 ± 0.05^*^	−0.13 ± 0.04	0.94 ± 0.01^***^	0.20 ± 0.04^***^	0.18 ± 0.05^***^	0.10 ± 0.04^*^	0.32 ± 0.04^***^
EW_AFE_	0.67 ± 0.20^**^	0.44 ± 0.11^***^		−0.15 ± 0.05	0.09 ± 0.05	−0.18 ± 0.05	0.52 ± 0.03^***^	0.10 ± 0.05	0.11 ± 0.06
ADG	−0.47 ± 0.42	−0.38 ± 0.09^*^	−0.49 ± 0.42		0.23 ± 0.04^***^	−0.16 ± 0.05	-0.06 ± 0.05	−0.18 ± 0.05	0.05 ± 0.05
MBW	0.25 ± 0.35	0.72 ± 0.01^***^	0.21 ± 0.20	0.39 ± 0.14^***^		-0.04 ± 0.04	0.17 ± 0.05^**^	0.04 ± 0.04	0.34 ± 0.04^***^
EN_12_	-0.54 ± 0.10^**^	-0.59 ± 0.13^**^	-0.27 ± 0.21	0.28 ± 0.38	0.72 ± 0.18^***^		-0.05 ± 0.05	0.81 ± 0.01^***^	0.11 ± 0.06
EW_12_	0.74 ± 0.29^*^	0.46 ± 0.20^*^	0.76 ± 0.08^***^	0.10 ± 0.22	0.24 ± 0.20	-0.52 ± 0.07^***^		0.41 ± 0.04^***^	0.13 ± 0.07
EM_12_	0.41 ± 0.20^*^	0.62 ± 0.20^**^	0.27 ± 0.22	0.11 ± 0.31	0.75 ± 0.25^**^	0.93 ± 0.03^***^	0.66 ± 0.13^***^		0.23 ± 0.05^***^
ADFI	0.27 ± 0.34	0.53 ± 0.20^**^	0.34 ± 0.19	-0.28 ± 0.29	0.51 ± 0.20^*^	0.40 ± 0.12^***^	0.61 ± 0.16^***^	0.55 ± 0.17^***^	
RFI	0.35 ± 0.47	0.06 ± 0.28	0.46 ± 0.21	-0.69 ± 0.30	-0.61 ± 0.19^**^	-0.44 ± 0.10^*^	0.45 ± 0.21^*^	-0.56 ± 0.18^**^	0.74 ± 0.02^***^
FCR	-0.44 ± 0.42	-0.48 ± 0.26	-0.41 ± 0.21	-0.18 ± 0.33	0.54 ± 0.19^**^	-0.74 ± 0.09^***^	0.82 ± 0.11^***^	-0.82 ± 0.03^***^	0.51 ± 0.16^**^
KLH-IgM	-0.39 ± 0.10^*^	-0.41 ± 0.10^*^	0.29 ± 0.23	0.31 ± 0.22	0.51 ± 0.18^**^	-0.37 ± 0.09^*^	-0.43 ± 0.11^*^	-0.35 ± 0.09^*^	0.47 ± 0.13^**^
NDV-IgG	-0.42 ± 0.12^*^	-0.35 ± 0.29^*^	-0.27 ± 0.13	0.29 ± 0.24	0.44 ± 0.16^*^	-0.49 ± 0.12^*^	-0.37 ± 0.09^*^	-0.43 ± 0.11^*^	0.52 ± 0.15^***^

^a^ ±SE = standard errors

^b^ AFE is age at first egg; BW_AFE_ is body weight at age at first egg; EW_AFE_ is egg weight at age at first egg; ADG is average daily gain; MBW is metabolic weight; EN_12_ is cumulative number of eggs 12 weeks from onset of lay; EW_12_ is average egg weight 12 weeks from onset of lay; EM_12_ is average daily egg mass 12 weeks from onset of lay; ADFI is average daily feed intake; RFI is residual feed intake; FCR is feed conversion ratio; KLH-IgM is natural antibody of IgM isotype binding to KLH antigen measured at 16 weeks; NDV-IgG is specific antibody IgG isotype binding to NDV antigen measured at 16 weeks of age.

***p < 0.001, ^**^ *p* < 0.01, ^*^ *p* < 0.05.

**TABLE 5:** Estimates of genetic^a^ (lower diagonal) and phenotypic^a^ (upper diagonal) correlations among production, efficiency and immunity traits during the laying period (12 weeks from on-set of lay).

Traits^b^	RFI	FCR	KLH-IgM	NDV-IgG
AFE	-0.06 ± 0.04	-0.08 ± 0.04	-0.01 ± 0.05	-0.02 ± 0.06
BW_AFE_	-0.05 ± 0.04	-0.01 ± 0.04	-0.08 ± 0.05	0.10 ± 0.06
EW_AFE_	0.08 ± 0.05	-0.09 ± 0.05	0.12 ± 0.06	-0.13 ± 0.05
ADG	-0.11 ± 0.05	-0.05 ± 0.04	0.10 ± 0.05	-0.06 ± 0.06
MBW	-0.04 ± 0.04	0.06 ± 0.04	0.09 ± 0.05	0.08 ± 0.06
EN_12_	-0.12 ± 0.09	-0.39 ± 0.02^**^	-0.07 ± 0.06	-0.02 ± 0.06
EW_12_	0.10 ± 0.05	0.41 ± 0.04^**^	-0.10 ± 0.05	-0.10 ± 0.05
EM_12_	-0.17 ± 0.05	-0.49 ± 0.02^**^ ^c^	-0.02 ± 0.05	-0.01 ± 0.06
ADFI	0.89 ± 0.01^***^	0.30 ± 0.05^**^	0.04 ± 0.05	0.03 ± 0.05
RFI		0.25 ± 0.07^**^	0.01 ± 0.05	0.15 ± 0.09
FCR	0.41 ± 0.10^**^		0.01 ± 0.06	0.07 ± 0.06
KLH-IgM	0.33 ± 0.15^*^	0.29 ± 0.24		-0.16 ± 0.13
NDV-IgG	0.50 ± 0.18^*^	0.34 ± 0.21	-0.49 ± 0.35	

^a^ ± SE, standard errors.

^b^ AFE, is age at first egg; BW_AFE_, is body weight at age at first egg; EW_AFE_, is egg weight at age at first egg; ADG, is average daily gain; MBW, is metabolic weight; EN_12_ is cumulative number of eggs 12 weeks from onset of lay; EW_12_ is average egg weight 12 weeks from onset of lay; EM_12_ is average daily egg mass 12 weeks from onset of lay; ADFI, is average daily feed intake; RFI, is residual feed intake; FCR, is feed conversion ratio; KLH-IgM, is natural antibody of IgM isotype binding to KLH, antigen measured at 16 weeks; NDV-IgG, is specific antibody IgG isotype binding to NDV, antigen measured at 16 weeks of age.

^***^ *p* < 0.001, ^**^ *p* < 0.01, ^*^ *p* < 0.05.

Genetic and phenotypic correlations of traits between the growing period and laying period are presented in Table 6. Highly negative genetic correlations were estimated between BW_12_ and AFE (r_g_ = −0.88; *p* < 0.01), and between ADG measured in both production periods (r_g_ = −0.91; *p* < 0.001). On the other hand, positive (0.62–0.82; *p* < 0.01) genetic correlations between two production periods were estimated for ADFI, KLH-IgM and NDV-IgG. Positive phenotypic correlation was estimated between MBW (r_p_ = 0.59; *p* < 0.001) and RFI (r_p_ = 0.55; *p* < 0.001) measured in both production periods.

**TABLE 6:** Estimates of genetic^a^ (r_g_) and phenotypic^a^ (r_p_) correlations for production, feed efficiency and immune traits between the growing and laying periods.

Growing period^b^	Laying period^b^	r_g_	r_p_
BW_12_	AFE	-0.88 ± 0.18^**^	-0.08 ± 0.05
ADG_1_	ADG_2_	-0.91 ± 0.12^***^	0.01 ± 0.05
MBW_1_	MBW_2_	0.20 ± 0.09^*^	0.59 ± 0.03^***^
ADFI_1_	ADFI_2_	0.80 ± 0.12^***^	0.01 ± 0.04
RFI_1_	RFI_2_	0.48 ± 0.32	0.55 ± 0.04^***^
FCR_1_	FCR_2_	0.19 ± 0.18	0.08 ± 0.04
KLH-IgM_1_	KLH-IgM_2_	0.62 ± 0.21^**^	0.05 ± 0.03
NDV-IgG_1_	NDV-IgG_2_	0.82 ± 0.17^***^	0.09 ± 0.06

^a^ ± SE, standard errors.

^b^ BW_12_, AFE, RFI, FCR, KLH-IgM, and NDV-IgG, are as described in Table 1: BW_12_, ADG_1_, MBW_1_, ADFI_1_, RFI_1_, FCR_1_, KLH-IgM_1_, and NDV-IgG_1_, are traits measured in the growing period; AFE, ADG_2_, MBW_2_, ADFI_2_, RFI_2_, FCR_2_, KLH-IgM_2_, and NDV-IgG_2_, are traits measured in the laying period.

***p < 0.001^, **^ *p* < 0.01.

## Discussions

The high heritability estimates for growth-related (BW_12_ and ADG) and feed efficiency (RFI and FCR) traits during the growing period indicate that these traits could be selected for in indigenous chicken to develop meat lines of high growth rates and that are feed efficient. The magnitude of heritability estimates for these traits suggest relatively higher prediction accuracies would be expected and therefore, mass selection could be utilized to improve growth and feed efficiency (Falconer and Mackay, 1996). Besides, higher additive genetic variance than residual variance for the respective traits, imply that relatively high genetic progress on growth and feed efficiency can be achieved through selective breeding. Post-maturity, the moderate to high heritability estimates for the production traits (AFE, EN_12_, EW_12,_ and EM_12_) and feed efficiency (RFI and FCR) suggest selection possibilities to breed for indigenous chicken egg lines that are early maturing, with high egg productivity and are feed efficient. Apart from average daily egg weight, higher residual variance than additive genetic variance for egg production and feed efficiency measures indicate that family selection would be a more reliable strategy to negate the residual environmental effects and improve accuracies of breeding values for these traits (Falconer and Mackay, 1996). Heritability estimates for production and feed efficiency traits in both production periods were within the range of values previously reported in local chicken in Tanzania (Lwelamira et al., 2009) but higher than those reported in commercial meat (Aggrey et al., 2010) and laying (van der Klein et al., 2015) chicken. Lower heritability estimates in the commercial population than the local chicken may be attributed to population differences. Studies by Aggrey et al. (2010) and van der Klein et al. (2015) used populations that have been subjected to intensive selection for production and feed efficiency and hence, are in general less diverse than the local chicken population.

Differences in heritability estimates for growth, metabolic weight, feed intake and feed efficiency traits between the growing period and the laying period may implicate physiological age as a source of variation. This could be in relation to gonadal hormones which are activated at point of sexual maturity and hence, influence body composition pre- and post-maturity (Loyd et al., 2011). Besides, Deeb and Lamont (2002) reported that sexual maturity coincides with the inflection of the growth curve, which corresponds to a shift in body composition away from protein accretion and towards fat deposition. Considering protein accretion is an energy expensive process than its maintenance while fat deposition requires less energy than its maintenance (Arthur et al., 2001), differences in body composition between pre- and post-mature age periods is likely to influence growth rate, maintenance requirement, feed intake and efficiency of feed use. Diet differences, in terms of energy density and protein content fed between the two production periods, may also be a contributing factor to variation in heritability estimates for these traits. In addition, considering that indigenous chicken are less active feeders (due to brooding behavior) during the laying period than during growing period (Dana et al., 2011), feeding behavior may also explain the differences in heritability estimates between the two production periods.

In immune traits, the moderate to high heritability estimates for KLH-IgM at 16 and 28−weeks of age indicate selection possibilities for natural antibodies to improve general immunity pre- and post-maturity. The advantage of using natural antibodies that bind to exo-antigens, such as KLH, to evaluate general immunity is that the study population did not and probably will not encounter KLH and therefore, reflects the capacity of innate immune function to respond to non-specific antigens not previously encountered (Star et al., 2007). Besides, KLH-IgM is significantly associated with survival in laying chicken and could be used as an accurate predictor of survivability in chicken (Sun et al., 2011). The low to moderate heritability estimates for NDV-IgG at 16 and 28 weeks of age indicate that genetic improvement of immune responses to vaccination against NCD pre- and post-maturity is feasible. Considering the study population encountered the NDV antigen *via* vaccination, presence of heritable variation for NDV-IgG could reflect an active status of memory immune cells specific to NDV antigen in both production periods (Walugembe et al., 2019).

Lower heritability and additive genetic variance estimates for the antibody traits (KLH-IgM and NDV-IgG) at 16 weeks than at 28 weeks of age suggests that relatively low prediction accuracies would be expected during the growing period compared to the laying period. In a commercial layer line divergently selected for natural antibodies at age 16 weeks for seven generations, Bovenhuis et al. (2022) estimated lower heritability and selection responses than expected in the high line compared to the low line. The study by Bovenhuis et al. (2022) attributed these observations to the effect of allele frequency changes in the *TLR*_*1*_ *A* gene polymorphism on genetic variance. The *TLR*_*1*_ *A* gene was previously identified on chromosome 4 and polymorphism of the gene was found as the most likely causal variant affecting the level of natural antibodies in chicken (Berghof et al., 2018). In this study, however, the experimental population was not subjected to artificial selection for antibody traits but rather production traits. Therefore, it is possible that either directional natural selection on immune-related genes at 16 weeks of age may be present in this population or that selection for body weight at week 12 of age may be having counter effects on antibody traits at 16 weeks of age. To this regard, there is need to carry out a genome wide association study in this study population to determine the genes that influence the antibody traits at 16 weeks of age and how allele frequencies of these genes are influenced by natural selection or by artificial selection for production traits. Conversely, higher heritability estimates for antibody traits measured post-maturity than pre-maturity may indicate effect of physiological age on immune function. Differences in physiological age has previously been linked to the development process of the immune function. For instance, Bernasconi et al. (2003) and Berghof et al. (2010) found that as animals advance in age, they encounter a variety of exogenous stimuli from either pathogens or environmental stressors which interact with genetic components to shape and enhance the formation of antibodies. Generally, in both production periods, higher residual variances than additive genetic variances for the antibody traits indicate lower repeatability for the immune measures and hence, response to selection would likely take a longer time, especially under mass selection. In this case, family selection coupled with improved management conditions (such as bio-security measures and vaccination) could optimize on accuracies of breeding value estimations while minimizing disease incidences (Farias et al., 2017).

Considering the study population is under selection for BW_12_ to develop meat lines, the positive correlation between BW_12_ and ADG indicate that improved body weight at 12 weeks will be associated with higher growth rates. Besides, BW_12_ is previously reported to have favorable genetic associations with hatch weight and market weight (24–26 weeks of age) in indigenous chicken (Ngeno et al., 2013). However, chicken of higher BW_12_ would have higher maintenance requirements during the growing period. Among the traits measured at the onset of lay, the genetic correlations indicated that early maturing birds had low mature body weight and low weight of first egg. The positive genetic correlation between BW_AFE_ and MBW suggest that chicken of low mature body weight are likely to have low metabolic body weights. In laying chicken, low mature body weight is mostly preferred as it is associated with low maintenance requirements and allows for more feed resources to be diverted towards egg production (Luiting, 1990). Knap and Rauw (2008), however, reports that focus to reduce maintenance requirement for the benefit of productivity compromises an individual’s adaptive capacity and physiological balance to cope with stressors within its production environment. Genetic associations between AFE and egg production traits suggest that age at first egg could be used as an indicator trait for cumulative egg numbers and average daily egg mass during the early laying period. Among the egg-related traits during the early laying period, chicken of high daily egg mass would be associated with high cumulative egg numbers but produce eggs of low weights (Dana et al., 2011). In both production periods, the highly positive genetic correlation of ADFI with production traits and metabolic body weight imply that a considerable proportion of the genetic variation in daily feed intake is associated with genetic differences in growth-related traits, egg-related traits and maintenance requirements (Yuan et al., 2015).

Feed conversion ratio was strongly correlated to ADG (growth period) and egg production traits than with ADFI, implying that variability in FCR was majorly influenced by production traits than by feed intake. On the other hand, the genetic and phenotypic correlations indicated that FCR efficient chicken consumed less feed but had higher growth rates during the juvenile period and higher egg production. Although FCR does not account for metabolic weight, the significant correlation with MBW suggest that efficient chicken had low maintenance requirement. These favorable associations imply that FCR is a trait that can be considered in breeding objectives intended to improve the efficiency of producing indigenous chicken during the growing and laying periods. Furthermore, FCR has extensively been used to measure feed efficiency due to its ease of computation and its direct association of costs and profits to quantities of feed (Aggrey et al., 2010). However, considering the strong genetic correlations between FCR and the production traits than with feed intake, Crews (2005) suggests that selection to reduce FCR may not necessarily be correlated specifically to improvements in efficiency, but may only reflect selection for increased productivity. Furthermore, FCR being a ratio trait, Aggrey et al. (2010) indicates that selection for such a trait may not translate into equivalent improvements in efficiency mainly because selection pressure may be disproportionately applied to the numerator or to the denominator (mostly in favor of the component trait with the most genetic variance). Besides, the confounding effects resulting from the relation between FCR and its component traits and the relation between its component traits, as observed in this study, prevent FCR from being an ideal measure of efficiency (Willems et al., 2013).

Due to the distributing properties of the regression procedure used to obtain RFI in this study, the efficiency measure was phenotypically independent from the production traits used in its estimation. Netter et al. (2004) reports that this phenotypic independence allows for comparison of individuals differing in the level of production traits. However, at a genetic level, Kennedy et al. (1993) found that genetic variability of RFI may not necessarily be independent of the production traits included in the model. In this study, genetic correlations between RFI and production traits indicated that feed efficient chicken during the growing period would be associated with higher growth rates while post-maturity, RFI efficient chicken would lay more eggs of low egg weights but have a higher egg mass. The magnitude of genetic correlation estimates was, however, higher between RFI with MBW than with production traits, an indication that RFI reflected more the variability in maintenance requirement than differences in production traits. Therefore, it is possible that the study population was diverting more feed resources to maintenance requirement rather than production requirement. Similar observations were previously reported by Aggrey and Rekaya (2013) in growing chicken and by Luiting (1990) in laying chicken. Besides, the negative genetic correlations between RFI and MBW in both production periods suggest that feed efficient chicken had a higher maintenance requirement. Aggrey et al. (2010) indicates that during the growing period protein accretion is at a higher rate than fat deposition due to muscle development and given that protein turn-over is an energy expensive process, could perhaps explain the high maintenance requirement in feed efficient chicken during this production period. This, however, implies that feed efficient growers will likely produce lean carcasses at market point. Similarly, the high maintenance requirement in laying chicken that were feed efficient may suggest that post-mature metabolic weight was majorly composed of protein tissues and hence, the use of more feed resources to maintain these tissues.

The positive genetic correlation between RFI and ADFI in both production periods indicate that improved efficiency would be accompanied by lower feed intake levels. Selection for feed efficiency measures is mostly aimed at reducing feed intake levels with a consequential effect on reduced feed costs, nitrogenous wastes and environmental footprint (Zhang and Aggrey, 2003). To this regard, higher genetic associations of ADFI with RFI than with FCR suggest that selection for RFI would be more beneficial in reducing feed intake than FCR-based selection. Besides, the positive correlation between RFI and FCR imply that improved RFI would also result in FCR efficient chicken. Considering that BW12 and AFE were not included in the estimation of RFI and FCR, presence of significant genetic and phenotypic correlations indicates that selection for body weight at week 12 and early sexual maturity would be accompanied with feed efficient meat and egg lines, respectively.

Among immune and production traits, the negative correlations between KLH-IgM and BW_12_, and between NDV-IgG and ADG implied immune deficiencies in chicken of high body weight at week 12 of age and growth rate during the juvenile period. At sexual maturity, the negative correlations between AFE and the antibody traits suggested that early maturing chicken had higher KLH-IgM and NDV-IgG levels. Similar observations by Zheng et al. (1998) indicated a physiological relationship between sexual maturation and development of the immune system exists, such that, earlier presence of circulating gonadal hormones positively influence the immune function. However, chicken of high mature body weights would be associated with low antibody levels. Similarly, genetic correlations between the antibody traits and egg-production traits indicated that chicken of high egg numbers, egg weight and egg mass had low KLH-IgM and NDV-IgG. Zerjal et al. (2021) reports that the immune system is heavily dependent on metabolic resources for proper functioning and therefore, it is expected to be in competition with other nutrient- and energy-demanding process, such as production, in an individual’s resource allocation strategy. This could explain the unfavorable associations between the antibody traits and production traits in both production periods and may indicate presence of biological trade-offs among these energy demanding functions. On the other hand, genetic correlations between the antibody traits with MBW, ADFI, and RFI in both production periods suggested that immune-competent chicken had higher maintenance requirements, consumed more feed and were feed inefficient. Considering that the development and maintenance of a fully competent immune system, as well as, the mobilization of immune responses to external stimuli have a metabolic cost (Rauw, 2012), it is possible that the study population was consuming more feed to support these immune functions rather than production and hence, the inefficiencies. Based on resource allocation theory, these observations indicate that an indirect relationship between levels (KLH-IgM) and activation (NDV-IgG) of humoral immunity and feed efficiency may be present.

Between the immune traits, the negative correlation indicated an antagonistic association between KLH-IgM and NDV-IgG. However, the lack of significance of the estimate suggests the association may not be significantly different from zero and the traits are probably genetically different. Antibodies binding to KLH and NDV represent different functional B cell activities and mechanisms underlying their formation vary with respect to the nature of the antigen (Parmentier et al., 2004; Star et al., 2007). For instance, KLH, a glycoprotein, is recognized by TLR-1 which is associated with Th-2 type of immune responses while NDV is a double stranded RNA recognized by TLR-3 which induces Th-1 type of immune responses (Cheng et al., 2014; Berghof et al., 2018). Mangino et al. (2017) indicates that the different TLRs and regulatory pathways are influenced by different genes and this could perhaps explain the genetic independence between KLH-IgM and NDV-IgG. Therefore, it is possible to simultaneously select for KLH-IgM and NDV-IgG to improve general immunity and specific immunity to NCD, respectively, with minimum to no adverse effects on either of the antibody traits.

For production traits measured pre- and post-maturity, the negative genetic correlation between BW_12_ and AFE implies that improved body weight at week 12 would lead to delayed sexual maturity. This may be undesirable for the egg lines given the negative associations between AFE and egg numbers (van der Klein et al., 2015). In ADG, the negative genetic correlation between the two production periods indicate that high growth rates during the juvenile period would result in low growth rates post-maturity. Growth curves of chicken follow a sigmoidal pattern in which point of growth inflection and attainment of asymptotic body weight coincides with point at sexual maturity (Ridho et al., 2021). Subsequently, post-maturity, growth rate tends to decelerate with increase in age and this could explain the observed genetic correlation between ADG measured pre- and post-maturity. Genetic associations between ADFI measured in both production periods suggests that feed intake in young and mature birds share a similar genetic background and therefore, chicken with high feed intake are likely to maintain this level of intake post-maturity.

Being a linear combination of production traits and metabolic weight, RFI aims to capture the variations among animals in energy utilization for production and maintenance requirement (Arthur et al., 2001). This ensures that the animals resulting from this form of selection would potentially be efficient both as producing individuals and breeding individuals. For instance, in developing feed efficient meat lines, it is expected that selection for RFI during the juvenile period will result in a breeding flock that will also be feed efficient when growth has virtually ceased and, maintenance and reproduction functions are a priority. Similarly, for egg lines, selected RFI efficient layers as the breeding flock are expected to produce progenies that can efficiently utilize feed resources for growth and development during the juvenile age to attain the required mature age and body weight. In this study, the positive genetic correlation between RFI measured pre- and post-maturity indicates that feed efficient chicken in the growing period would maintain their efficiency during the laying period. However, the non-significance level of the correlation estimate suggests that RFI determined prior to sexual maturity may not be an accurate predictor of post-maturity RFI. In this case, low RFI growers may or may not rank as efficient breeders and low RFI layers in the breeding flock may or may not produce progenies that efficiently utilize feed resources to attain expected sexual mature age and weight.

As animals grow, composition of their gain shifts from protein accretion to fat deposition with a substantial shift occurring around the time of sexual maturity and hence, variability in body composition between young and mature animals (Arthur et al., 2001). This could be supported by the low genetic correlation in MBW measured between the two production periods indicating that genetic influence of maintenance requirements at juvenile ages may not necessarily be same requirements post-maturity. Since energy expenditure associated with deposition and maintenance of tissues varies between protein and fat tissues (Loyd et al., 2011) and that MBW is a major contributor to variation in RFI (Luiting, 1990), the efficiency of feed use is expected to vary depending on the body composition. Therefore, changes in body composition associated with advancing physiological maturity could partially explain the low genetic correlations between pre- and post-maturity RFI. Besides, Luiting (1990) also reports that genetic differences between RFI measured pre- and post-mature periods could be related to differences in feeding behavior, physical activity, nutrient digestibility, heat increment, and energy homeostasis and partitioning. On the other hand, during the juvenile period the focus of selection for meat lines is to maximize on growth and development processes occurring during this period while post-maturity the focus is to have early maturing chicken with high egg production. These production functions contribute differently to variation in feed consumed (Zerjal et al., 2021) and therefore, the efficiency of feed utilization in the respective production periods is expected to vary. Higher phenotypic correlation than genetic correlation for RFI and MBW could suggest the traits measured in the two period share common environmental effects (Falconer and Mackay, 1996). This indicates that if favorable environmental conditions are experienced in both production periods, feed efficient chicken and those of low maintenance requirement during the growing period are likely to maintain the same performance during the laying period (Willems et al., 2013). Therefore, high producing individuals selected to be breeders should perform in the same environment to maintain the similar efficiency and maintenance requirement.

The strong and positive genetic correlations between antibody traits measured at 16 and 28 weeks of age could indicate that KLH-IgM and NDV-IgG share a common genetic background at juvenile age and at maturity. Similar observations by Bovenhuis et al. (2022) in laying chicken found that genetic correlations between natural antibodies measured at 16 and 32 weeks of age were not significantly different from one. Despite the strong genetic correlations, differences in heritability estimates of antibody traits between the two age periods suggest that environment-related effects have more influence than additive genetic effects (Bovenhuis et al., 2022). Boa-Amponsem et al. (1997) indicates that the regression of the bursa of Fabricius (production site of B cells) leading into maturation and differentiation of the immune system mostly occurs when birds are approaching maturity (9–20 weeks) and immediately post-maturity (20–40 weeks) and that these processes are influenced by similar genes. Therefore, chicken with higher levels of KLH-IgM and NDV-IgG at juvenile age are likely to rank as immune-competent at maturity. This implies that early selection for KLH-IgM and NDV-IgG at 16 weeks of age is feasible and may be effective on immune function at 28 weeks of age. Besides, presence of a linear association between immune functions and age (Berghof et al., 2010) indicate that selected individuals at 12 weeks of age will likely have enhanced antibody responses even at advanced ages, that is, beyond 28 weeks of age. This is based on the idea that environmental sensitization from exogenous stimuli an individual may encounter over time shapes and enhances antibody responses (Bernasconi et al., 2003). For instance, in an egg line, Sun et al. (2013) estimated positive correlations between KLH-IgM measured at 20 and 40 weeks of age and between KLH-IgM measured at 40 and 65 weeks of age, indicating a peak in antibody levels between ages 20–40 weeks, followed by a stable period until 65 weeks of age.

## Conclusion

The low to high heritability estimates for growth-related, egg-related, feed efficiency and antibody traits measured during the growing and laying periods indicate presence of heritable variation that could be exploited to improve production, feed efficiency and immunity in indigenous chicken. Genetic correlations among the traits suggest that improved feed efficiency would be associated with high growth rates, early maturing chicken, high egg mass and reduced feed intake. In contrast, improved general (KLH-IgM) and specific (NDV-IgG) immunity would result in lower growth rates and egg mass but associated with early sexual maturation and high feed intake. Unfavorable genetic correlations between feed efficiency and immune traits imply that chicken of higher productivity and antibody levels will consume more feed to support both functions. These associations indicate that selective breeding for feed efficiency and immune-competence may have genetic consequences on production traits and should therefore be accounted for in indigenous chicken improvement programs.

## Data Availability

The raw data supporting the conclusion of this article will be made available by the authors, without undue reservation.
